# The early evolutionary landscape of osteosarcoma provides clues for targeted treatment strategies

**DOI:** 10.1002/path.5699

**Published:** 2021-05-25

**Authors:** Michal Kovac, Baptiste Ameline, Sebastian Ribi, Monika Kovacova, William Cross, Maxim Barenboim, Olaf Witt, Stefan Bielack, Andreas Krieg, Wolfgang Hartmann, Michaela Nathrath, Daniel Baumhoer

**Affiliations:** ^1^ Bone Tumor Reference Centre Institute of Medical Genetics and Pathology, University Hospital Basel, University of Basel Basel Switzerland; ^2^ Faculty of Informatics and Information Technologies Slovak University of Technology Bratislava Slovakia; ^3^ Evolution and Cancer Laboratory Barts Cancer Institute, Barts and the London School of Medicine and Dentistry, Queen Mary University of London, Barbican London UK; ^4^ Department of Pediatrics and Children's Cancer Research Center Klinikum rechts der Isar, Technical University of Munich, School of Medicine Munich Germany; ^5^ Hopp Children's Cancer Center Heidelberg German Cancer Research Center and University Hospital Heidelberg Heidelberg Germany; ^6^ Klinikum Stuttgart – Olgahospital, Stuttgart Cancer Center Stuttgart Germany; ^7^ Paediatric Orthopaedic Department University Children's Hospital Basel Basel Switzerland; ^8^ Division of Translational Pathology Gerhard‐Domagk‐Institut of Pathology, University Hospital Münster Münster Germany; ^9^ Department of Pediatric Oncology Klinikum Kassel Kassel Germany

**Keywords:** osteosarcoma, molecular diagnostics, targeted therapies, personalized medicine, cyclin‐dependent kinase inhibitors, cyclins, BRCAness, PARP inhibitors

## Abstract

Osteosarcomas are aggressive primary tumors of bone that are typically detected in locally advanced stages; however, which genetic mutations drive the cancer before its clinical detection remain unknown. To identify these events, we performed longitudinal genome‐sequencing analysis of 12 patients with metastatic or refractory osteosarcoma. Phylogenetic and molecular clock analyses were carried out next to identify actionable mutations, and these were validated by integrating data from additional 153 osteosarcomas and pre‐existing functional evidence from mouse PDX models. We found that the earliest and thus clinically most promising mutations affect the cell cycle G1 transition, which is guarded by cyclins D3, E1, and cyclin‐dependent kinases 2, 4, and 6. Cell cycle G1 alterations originate no more than a year before the primary tumor is clinically detected and occur in >90% and 50% of patients of the discovery and validation cohorts, respectively. In comparison, other cancer driver mutations could be acquired at any evolutionary stage and often do not become pervasive. Consequently, our data support that the repertoire of actionable mutations present in every osteosarcoma cell is largely limited to cell cycle G1 mutations. Since they occur in mutually exclusive combinations favoring either CDK2 or CDK4/6 pathway activation, we propose a new genomically‐based algorithm to direct patients to correct clinical trial options. © 2021 The Authors. *The Journal of Pathology* published by John Wiley & Sons, Ltd. on behalf of The Pathological Society of Great Britain and Ireland.

## Introduction

Osteosarcoma is the most common primary malignant tumor of bone that generally develops in children and adolescents, but there is a large population of patients presenting the disease in the last decades of life [1]. The osteosarcoma's cell of origin is currently unknown, but it is speculated that these tumors originate from early osteoblastic progenitors [2] that might be vulnerable to chromoanagenesis [3]. Should this happen, most cells will die in apoptosis. However, in rare cases, chromoanagenesis can generate *TP53* mutations that allow cells with rearranged genomes to enter mitosis and eventually escape programmed cell death [4]. However, osteosarcomas can also develop without *TP53* mutations and recent studies suggested that its mutations occur across a much broader range of genes (67 according to one [5]), questioning how such genetic diversity contributes to the development of a single cancer entity. In this study, we attempted to tackle the above conundrum by analyzing longitudinal sequencing data from matched primary and recurrent tumors of 12 patients. Phylogenetic and molecular clock analyses were then performed to estimate the timing of different cancer driver mutations and we explored which of the initiating ones can be utilized as therapeutic targets. These data provide an understanding of the evolutionary trajectories underpinning the development of osteosarcoma and highlight cell cycle G1 mutations as particularly promising early actionable mutations that become clonally pervasive in metastatic osteosarcomas.

## Materials and methods

### Patients and samples

The study was approved at the University Hospital Basel, following the approval of the ethical committee for mutational analysis of anonymized samples (‘Ethikkommission beider Basel’ ref 274/12). Informed consent was obtained from all 12 patients. All tumor samples were evaluated by an experienced bone pathologist to confirm the diagnosis. Tumor histology and all other clinically relevant characteristics were retrieved at this stage and are depicted in supplementary material, Table S1.

### Validation cohorts

The validation cohort comprised two independent mutation datasets: the International Cancer Genome Consortium dataset consisted of mainly untreated primary osteosarcomas (*n* = 112) [5], whereas the INFORM dataset included lung metastases (*n* = 41) [6]. The mutation data used in our study had already been processed and therefore no additional filtering was needed.

### Sequencing data availability

Illumina sequencing data have been deposited in the European Nucleotide Archive under the study accession number EGAS00001005199.

### Illumina sequencing and variant calling

Illumina sequencing was performed on HiSeq 4000 sequencers (Illumina, San Diego, CA, USA) using the standard protocols for whole exome (following sequence capture with an Agilent SureSelect Human Exome Capture kit, ver. 4; Agilent, Santa Clara, CA, USA) and whole genome sequencing. Raw reads were quality‐checked (fastqc ver. 0.11.7, https://www.bioinformatics.babraham.ac.uk/projects/fastqc/), adapter‐trimmed, duplicate‐removed (Picard tools ver. 2.9, https://broadinstitute.github.io/picard/), and mapped onto the hs37d5 version of the human genome (BWA ver. 0.7). The GATK pipeline ver. 3.8 (https://gatk.broadinstitute.org/hc/en-us) was used for variant calling. Raw variants were filtered, and high‐quality alleles extracted using the following settings: QD > 10.0, MQ > 40.0, FS < 30.0, SOR < 3.0, MQRankSum > −12.5, and ReadPOsRankSum > −8.0. Somatic variants were determined based on their absence in the matching tumor‐free tissue. Variants that passed these filters were annotated using ANNOVAR databases (ver. 2019). Putative pathogenic variants were identified based on the information from CLINVAR and COSMIC databases or by passing the following *in silico* criteria: SIFT < 0.05, PolyPhen2 > 0.7, Mutation Taster > 0.7, GERP++ > 0, CADD > 10, and PhyloP VT/PL > 0. Indels were identified using the Scalpel tool (http://scalpel.sourceforge.net) and then their presence was verified in the GATK data and by visual inspection in the IGV browser. Copy‐number information was determined using Nexus Discovery software, version 10 (BioDiscovery, El Segundo, CA, USA), from whole genome sequencing data. Structural rearrangements were visualized with the RCircos package (https://cran.r-project.org/web/packages/RCircos/index.html) after being confirmed with the BreakDancer algorithm (http://breakdancer.sourceforge.net). Sequencing summary statistics are depicted in supplementary material, Table S2.

### Fusion‐gene identification from RNA sequencing data

In patients P6 and P7, RNA sequencing libraries were prepared from tumor DNA using the TruSeq RNA Sample Preparation Kit v2 (Illumina). Total RNA was extracted from fresh‐frozen tumor tissue and mRNA was then purified from 1 μg of total RNA using oligo(dT) beads. Paired‐end sequencing was performed on the Illumina HiSeq 2500 sequencers in rapid run mode according to the manufacturer's protocol using the TruSeq SBS Kit (ver. 3). Sequencing reads were mapped onto the GRCh37 human reference genome using STAR or HISAT2 (http://daehwankimlab.github.io/hisat2/). ChimeraScan (https://bioconductor.org/packages/release/bioc/html/chimera.html), defuse (https://github.com/amcpherson/defuse), and FusionCatcher (https://github.com/ndaniel/fusioncatcher) algorithms were used to detect chimeric RNA transcripts from fastq data. Predicted fusions were filtered out based on the presence of chimeric reads, which were ‘blasted’ against the human transcriptome to exclude further ambiguity concerning the involved partners.

### Semiconductor sequencing

The ion torrent sequencing (Thermo Fischer Scientific, Waltham, MA, USA) approach was taken for technical replication of selected somatic variants. Used in conjunction with the AmpliSeq library construction kit, the Ion AmpliSeq comprehensive cancer panel was used to capture exons of 409 genes from the Cancer Gene Census database and the resulting libraries were run on the Ion PGM sequencers. Raw reads were processed using the Ion reporter software with standard settings.

### Homologous recombination deficiency (HRD) testing

Homologous recombination repair deficiency (HRRD) was classified based on mutational signature analysis that was carried out using EMu software (https://github.com/andrej-fischer/EMu). In addition, the degree of genomic scarring indicative of HRRD was determined as the sum of the degree of loss of heterozygosity (LOH) [7], telomeric allelic imbalance (TAI) [8], and large‐scale transitions (LSTs) [9]. Consequently, the HRD‐LOH score counts the number of LOH regions longer than 15 Mb. HRD‐TAI similarly determines the prevalence of more than 11‐Mb‐long allele‐imbalanced regions that extend to sub‐telomeres but do not cross the centromere. Finally, the HRD‐LST score is defined as the number of break points between regions longer than 10 Mb but after filtering out regions shorter than 3 Mb and adjusting for sample ploidy. Osteosarcomas with a summary score of 42 or higher were deemed positive. XY chromosomes were omitted from the analysis.

### Phylogenetic analysis

We built phylogenetic trees from the SNV data using PAUP software (http://phylosolutions.com/paup-test/). First, we converted each variant set into a binary matrix, where the rows related to a particular tumor or the normal sample and the columns related to a specific variant. The binary encoding (0/1) designated the absence or presence of a variant. A nexus file was used to specify the parsimony parameters needed for the tree construction along with the variant matrix. The following functions and parameters were used: (1) the outgroup function was used to root all resulting trees to the normal sample (effectively, a column on the mutation matrix containing only zeros); (2) the hsearch function was used to perform a heuristic search of 10 000 000 trees from the given tree space, with 1000 of the shortest trees output for the main analysis; (3) the bootstrap function was used to perform a sub‐sampling procedure 10 000 times that involved randomly selecting a set of mutations from the binary matrix (with replacement), with the proportion of each branch instance reported in a log file; and (4) the allTrees function was used in all cases because fewer than ten samples were present. This made it possible to perform an extended ‘brute‐force’ run to acquire shortest tree(s) from the total search space, at the expense of computational time. The homoplasy index for the most parsimonious tree in a given set was automatically calculated and output to the PAUP log file. To obtain the shortest, and thus most parsimonious, tree, an Rscript using the ape package was used to input the .tre file.

### Fluorescence *in situ* hybridization

CDK4 amplifications were determined using fluorescence *in situ* hybridization, which was carried out on formalin‐fixed, paraffin‐embedded slides using the standard procedure specified by the probe manufacturer (ZytoLight® SPEC CDK4/CEN 12 Dual Color; supplier: LabForce AG, Muttenz, Switzerland). Hematoxylin and eosin staining was carried out in parallel for comparative purposes.

### Statistical analyses

Statistical analyses were performed in R. Unless otherwise stated, all statistical comparisons of two distributions used (wherever possible paired) Welch's *t*‐test, and 95% confidence intervals (CIs) of means were determined using the ci function of the gmodels package in R. Contingency tables were analyzed using Fisher's exact test.

## Results

### The mutation landscape of primary and recurring osteosarcomas

We performed paired exome and low‐coverage genome sequencing of chemotherapy‐naive osteosarcoma primaries (P) and local and/or distal recurrences (REC) in ten patients. In two additional patients, primary tumors before and after neoadjuvant chemotherapy were sequenced, but recurring tumors were not available for the analysis. In total, our discovery set comprised 36 tumor exome–genome pairs, which were sequenced to a median depth of 156X (range 87–420X) and 20X (range 5–77X), respectively. In each patient, additional exomes and genomes were sequenced using blood‐derived leukocyte DNA to the equivalent median depth of paired tumor samples.

**Figure 1:**
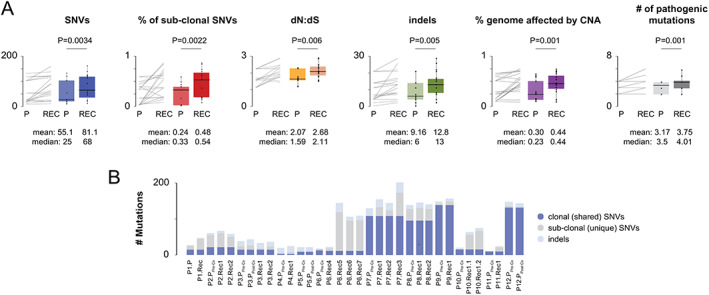
Genetic diversity in primary and recurring osteosarcoma. (A) Osteosarcoma recurrences acquire more somatic SNVs and indels and have a higher proportion of genome affected by copy‐number alterations. A paired comparison of mutation burden between primary and recurring osteosarcomas is shown by line and box plots. The box represents the upper and lower 25% quantile; the central line represents the median value. *P* values were calculated using paired two‐sample *t*‐tests and are shown above the plots. Mean and median values are shown below the plots. (B) Total mutation burden for each tumor is shown as stacked bars.

For the discovery of somatic mutations, we restricted our analysis to protein‐coding regions. Primary tumors acquired a median of 57 single‐nucleotide variants (SNVs, range 6–144, 95% CI = 26.8–88.4) and 10 indels (range 0–21, 95% CI = 6.91–13.5), whereas recurrent tumors samples had ~50% more of each type of mutation (Figure 1). Without exceptions, the C:G>T:A changes were the most common changes followed by C:G>A:T and T:A>C:G changes. To prioritize these mutations for further investigation, we excluded all SNVs with moderate or benign functional effects (i.e. SIFT > 0.05, PolyPhen2 < 0.7, Mutation Taster < 0.7, GERP++ < 0, CADD < 10, and PhyloP VT/PL < 0) but kept all protein truncating and splice‐site mutations. We then inspected all variants in the Integrated Genome Viewer to exclude any variant of evidently poor quality and merged the resulting list of variants with copy‐number alterations (gains of 5+ copies or losses of both copies) that were identified simultaneously. Eventually, mutations in 29 cancer driver genes remained, of which about one quarter were functionally relevant for cell cycle G1 progression.

**Figure 2:**
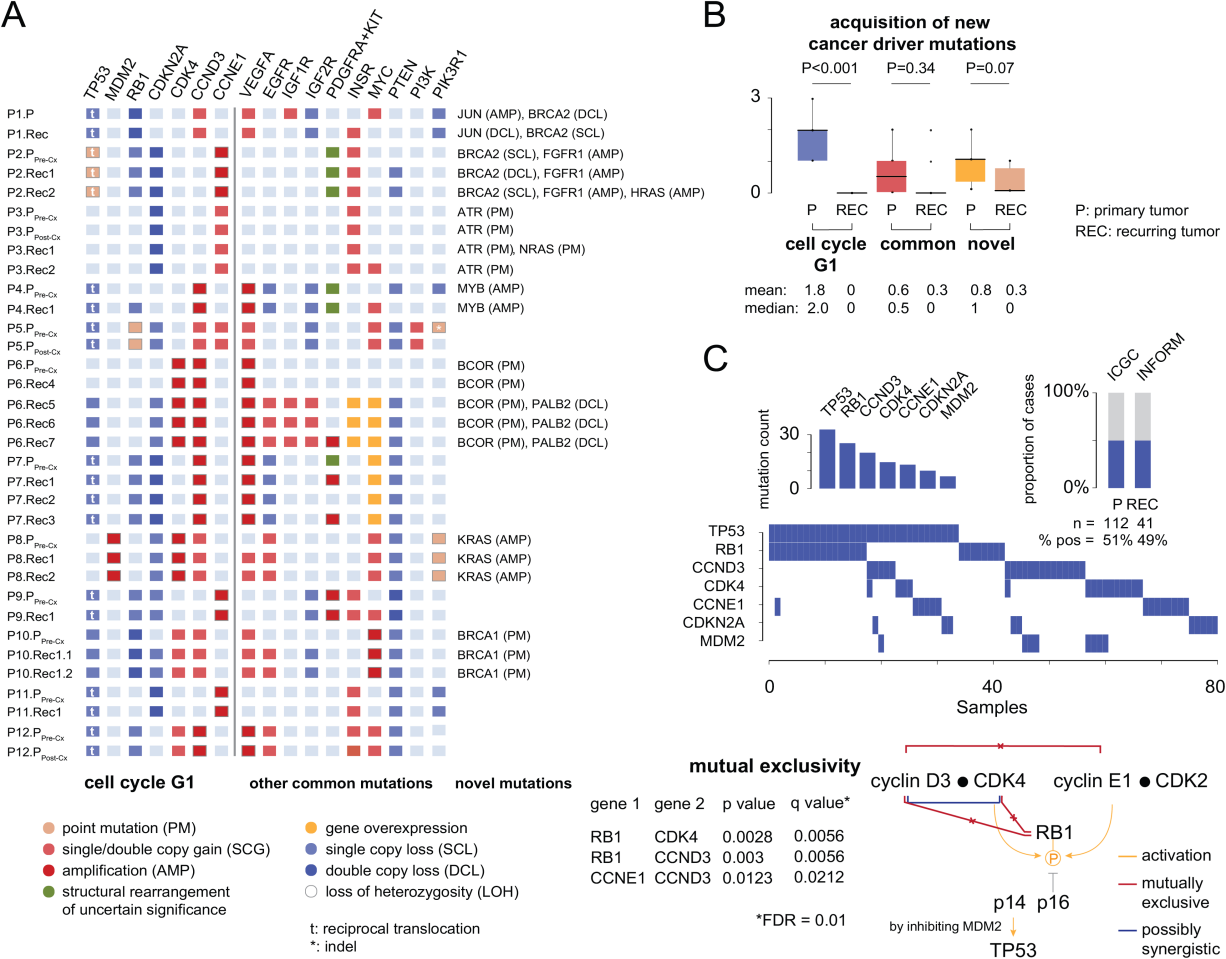
Cancer driver mutations. (A) Cancer driver genes were grouped according to their known relation to osteosarcoma pathogenesis. For each gene and each tumor, different types of mutations are shown. (B) The earliest, the most pervasive, and thus clinically the most relevant mutations affect cell cycle G1 transition and are acquired prior to the clinical manifestation of the primary tumor. The box represents the upper and lower 25% quantile; the central line represents the median value. *P* values were calculated using paired two‐sample *t*‐tests and are shown above the plots. Mean and median values are shown below the plots. (C) Mutual exclusivity of cell cycle G1 mutations was validated using data from two independent studies, comprising both primary (ICGC study [5]) and metastatic osteosarcomas (INFORM study [6]). The proportion of tumors harboring mutations and the individual mutation frequencies are shown as stacked bars. The mutual exclusivity of cell cycle G1 mutations is shown below and was tested according to Canisius *et al* [10]. The corresponding *p* and *q* values are shown below and were calculated under the assumption of a false discovery rate of 0.01.

Cell cycle G1 progression normally occurs when favorable conditions are met and is dependent on the activation of cyclin‐dependent kinases (CDK2, 4, 6) by their regulatory units called cyclins. When the cell is ready to divide, Rb protein is phosphorylated to pRb by CDKs, leading to the inactivation of Rb. This process allows a cell to enter into the cell cycle S state that is normally repressed by p16, which is encoded by *CDKN2A*. *CDKN2A* also encodes p14ARF that controls the MDM2–p53 pathway and DNA integrity checkpoints, which would normally prevent a cell that underwent chromoanagenesis from entering mitosis. However, this does not seem to be the case for our patients because *MDM2*/*TP53* mutations were present in more than half of the cases (7/12, 58%; Figure 2 and supplementary material, Figure S1), followed by mutations affecting *CDKN2A* (4/12, 33%), *RB1* (3/12, 25%), cyclin D3 (*CCND3*, 3/12, 25%), and cyclin‐dependent kinase 4 (*CDK4*, 2/12, 16%).

From this list, we selectively validated *CDK4* amplifications using fluorescence *in situ* hybridization (FISH) and confirmed that cells carrying high‐level gains were ubiquitously present in all analyzed tumor sections (supplementary material, Figure S2). We further noted that *CDK4* mutations and dual‐copy deletions of *RB1* were mutually exclusive, as were mutations affecting genes encoding RB1 and CCND3. In three cases, *CDKN2A* deletions complemented *CCNE1* amplifications (3/12, 25%), suggesting an independent activation of Rb via the CDK2 pathway.

To replicate our findings, we analyzed public mutations data from two previous studies [5, 6], yielding a cell cycle G1 mutation prevalence that was significantly lower than that observed in the discovery set of tumors (P: *n* = 58/112, 51.7%; REC: *n* = 20/41, 48.7%). We did not find any specific attribute that would explain the difference, although we initially thought that a bias for adult osteosarcomas in Behjati *et al*'s cohort [5] could be the case. However, we still confirmed the mutual exclusivity of *CCND3* and *CCNE1* amplifications in these patients, as well as the mutual exclusivity of *CDK4* amplifications and loss‐of‐function mutations in *RB1* (Figure 2C). The cumulative prevalence of *CCNE1*, *CCND3*, and *CDK4* amplifications in tumors with active CDKs was 60.2% (*n* = 47/78), which translates into 30.7% (*n* = 47/153) of all cases.

### Phylogenetic analysis confirms early acquisition of cell cycle G1 mutations

**Figure 3:**
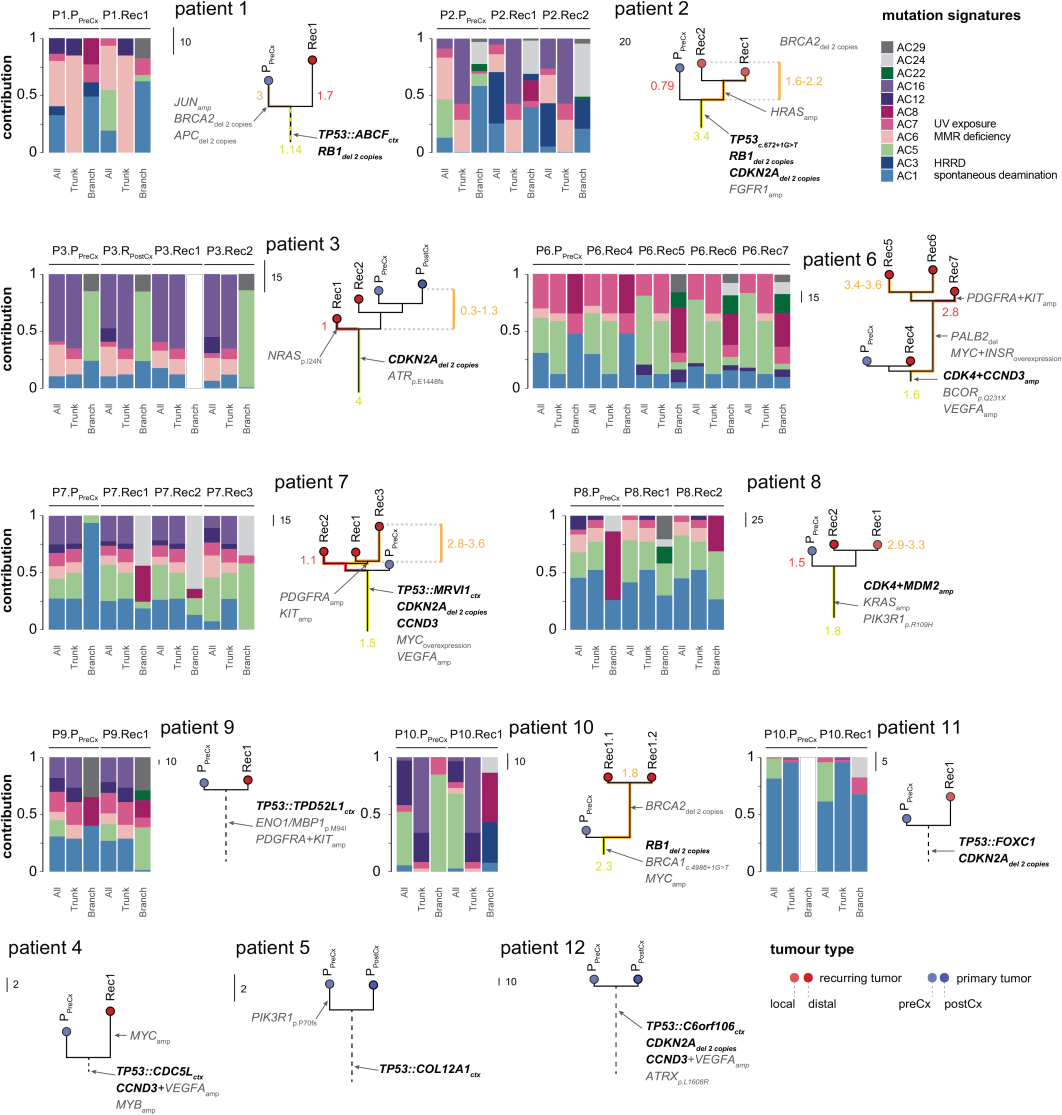
Phylogenetic analysis and mutational signatures. Maximum‐parsimony trees were constructed from exome SNVs. Cell cycle G1 mutations are depicted in bold, illustrating their enrichment on the trunks. The ratio of non‐synonymous to synonymous mutations is shown on selected phylogenies: values show the ratio of the non‐synonymous and synonymous SNVs across trunks (T_ns/s_) and branches (B_ns/s_). A higher ratio is indicative of an increased number of non‐synonymous mutations and therefore potentially also of selective benefits. Mutational signature analysis is shown next: stacked bar plots show the relative contribution of individual mutation signatures, split between mutations occurring at the phylogenetic trunk and branches/leaves. Mutational signatures are not shown for tumors that acquired fewer than ten somatic SNVs. amp, amplification; ctx, chromosomal translocation; del, deletion; HRRD, homologous recombination repair deficiency; MMR, mismatch repair of DNA.

Cell cycle G1 mutations were chosen for more detailed analyses based on their known therapeutic potential, good population prevalence, and almost universal pervasiveness across recurrent tumor manifestations. To determine if other mutations would follow the same pattern, we constructed maximum‐parsimony trees and inspected their topologies (Figure 3 and supplementary material, Table S3). Using somatic SNV data, we observed that about half of the trees were characterized by shorter trunks (variants ubiquitous across different tumors) with comparatively longer branches, thus appearing ‘apple tree’‐shaped. Mutations affecting cell cycle G1 were located on the trunks, whereas somatic mutations affecting other pathways occurred equally on trunks, branches, and leaves. For example, the *NRAS* p.I24N mutation (P3), which is a well‐proven gain‐of‐function mutation, was detected only in a single metastasis that was phylogenetically ancestral to the primary tumor and to another metastasis, neither of which had it. Similarly, amplifications of *MYC*, *BRCA2*, and a co‐amplification of the region *PDGFRA–KIT* occurred mostly in recurring tumors (P1, P2, P4, P6, P7, P10), whereas *JUN* and *APC* mutations occurred in a single osteosarcoma primary (P1). Finally, the most interesting novel somatic mutations that occurred on trunks and branches were activating mutations of the RAS GTPases *KRAS* and *HRAS* that predispose to non‐ossifying fibromas [11] and malignant giant cell tumors of the bone [12, 13] but have not been reported in osteosarcoma thus far.

We therefore hypothesized that the selective benefits of mutations occurring on branches and leaves were comparably weaker for the mutations occurring on trunks and searched for evidence by examining the relative ratio of non‐synonymous to synonymous mutations at the corresponding trunks and branches (Figure 3 and supplementary material, Table S4). The non‐synonymous to synonymous mutation ratio is normally used to estimate the balance between neutral mutations, purifying selection, and beneficial mutations, such that values that are significantly above 1 were unlikely to occur without at least some of the mutations being advantageous. This indeed proved to be the case for most mutations occurring on a trunk, whereas the reduction in positive values on branches and leaves indicated weakened selection pressure.

### Chromothripsis and staircase amplifications generate cell cycle G1 mutations

**Figure 4:**
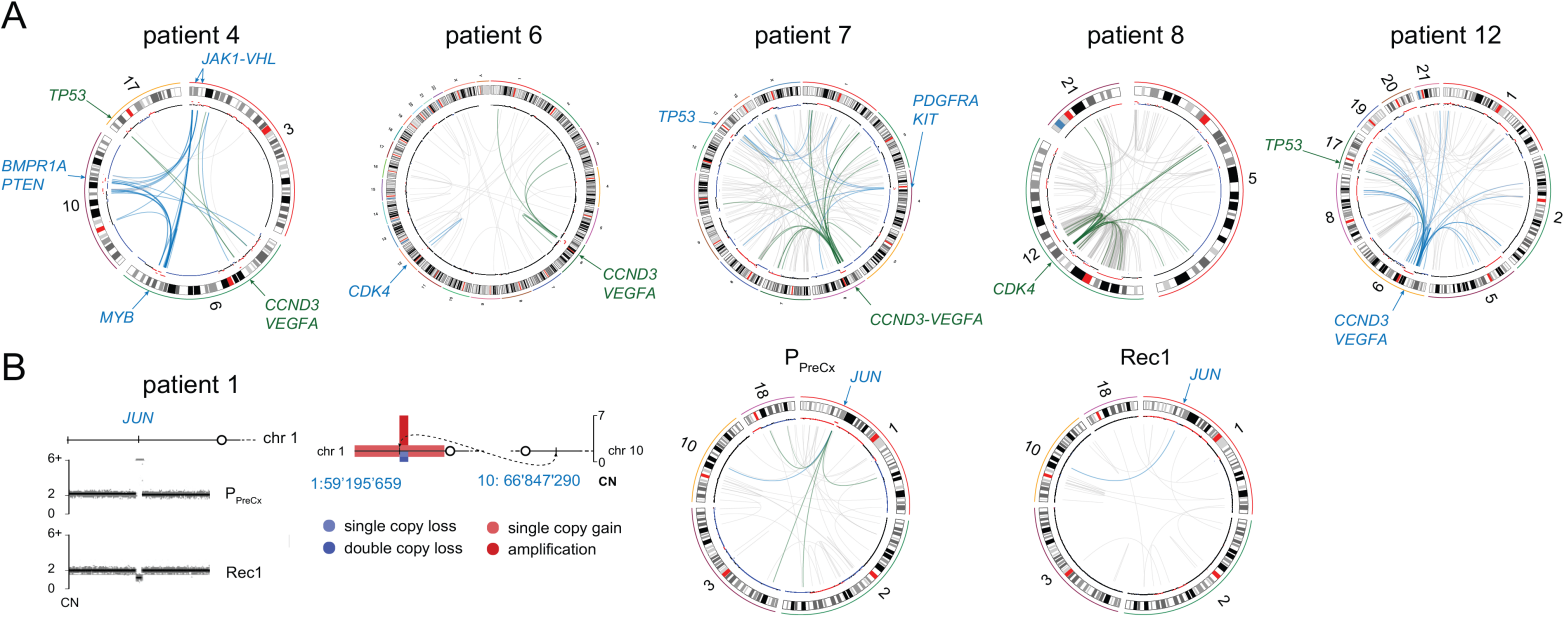
Complex structural alterations. (A) Cell cycle G1 mutations are often generated by repeated breakage of the chromosomal centromere, followed by rejoining with multiple chromosomes. Because the same structural pattern is detected across all sampled tumors, we assumed that this process takes place before the metastatic cells diverge. Five examples of genomic profiles of primary tumors are displayed. The circular representations of a portion of tumor genomes depict genomic copy numbers in the inner outer track. The dots represent the log_2_ values of normal (black), lost (blue), and gained (red) regions. Chromosomal translocations are depicted as lines between the corresponding positions. (B) An example of the breakage and reunion mechanism that generated *JUN* amplification in the primary tumor of patient P1. A lung metastasis, which was resected 2 years later, shows the loss of both chromosomal copies of the gene and no extra chromosomal rearrangements, which were detected in the primary tumor.

Primary osteosarcomas typically had ~30% of the genome affected by copy‐number alterations (CNAs), which was ~14% less than in matched recurring tumors (P: 95% CI = 21–40%; REC: 95% CI = 35–53%). Most CNAs were either single copy gains or losses but about ~1% of the cancer genomes underwent complex reorganization (Figure 4A), resulting in chromothripsis and centromere/telomere‐driven staircase amplifications. Chromothripsis (CT) events were larger and affected ~389 genes (95% CI = 0–783 genes) across a 32 Mb chromosomal sequence (95% CI = 4.7–94.1 Mb). Staircase amplifications (SAs) were comparably smaller, affecting either ~35 genes within the ~3.5 Mb sequence flanking the ends of the chromosomes (*n* = 6, 95% CI = 0.1–7.7 Mb, 3.9–62.4 genes) or ~103 genes within the ~8.5 Mb sequence flanking the centromeres (*n* = 14, 95% CI = 6.7–17.3 Mb, 79–206 genes).

**Figure 5:**
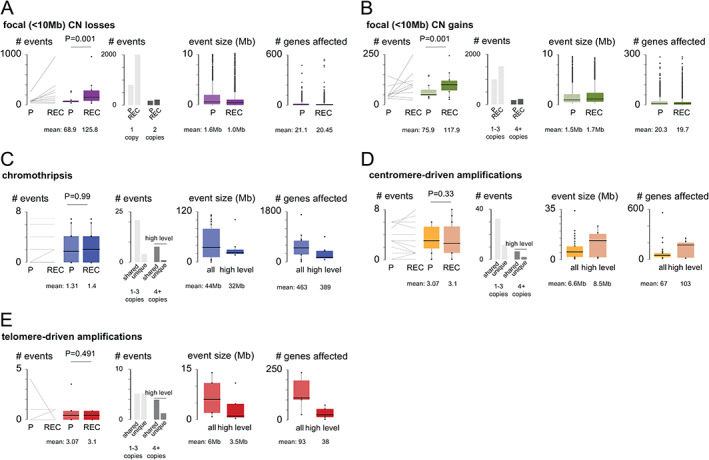
Numerical and structural alterations. A paired comparison of CNA burden between primary and recurring osteosarcomas is shown by line and box plots. The box represents the upper and lower 25% quantile; the central line is the median value. *P* values were calculated using paired two‐sample *t*‐tests and are shown above the plots. Mean and median values are shown below the plots. Note that most complex rearrangements occurred early in evolutionary time, whereas focal gains and losses were more prevalent in recurring tumors.

We specifically searched for CT/SAs affecting genes involved in cell cycle G1 progression [*CDK4* (*n* = 2), *CCND3* (*n* = 4), *CCNE1* (*n* = 3)] or/and related functions [*MYB* (*n* = 1), *KIT* (*n* = 3), *VEGFA* (*n* = 4), and *PDGFRA* (*n* = 3)] and noted that they were 5–7 times more frequent in the primary tumors (Figure 5). Conversely, focal CNAs were more frequent in recurring tumors (focal gains: P: mean number of events = 75.9, 95% CI = 39–112; REC: mean = 117.9, 95% CI = 55.3–137, *p* < 0.001, paired two‐sample *t*‐test; focal losses: P: mean = 68.9, 95% CI = 31.2–86.4; REC: mean = 125.8, 95% CI = 13.9–215, *p* < 0.001, paired two‐sample *t*‐test). To define these events, we used the common cytogenetic threshold of 10 Mb [14] and found that most focal alterations were in fact only 1–1.5 Mb wide, affecting 20 genes on average (focal gains: P: mean number of events = 20.33, 95% CI = 9.91–30.02; REC: mean = 19.7, 95% CI = 8.45–31.1; focal losses: P: mean = 21.1, 95%CI = 10.7–33; REC: mean = 20.45, 95% CI = 11.1–29.8). Focal bi‐allelic deletions were of a smaller kind, removing only up to eight genes (P: mean = 8.18 genes, 95% CI = 0.01–16.2; REC: mean = 4.84 genes, 95% CI = 0.01–10.9) and functionally serving as ‘second hits’ in tumor suppressor genes (*APC*, *BRCA2*, *BMP2*, *NRAS*, *VHL*, *PTEN*). Focal amplifications were slightly larger (P: mean = 28 genes, 95% CI = 13.2–36.5; REC: mean = 32.9 genes, 95% CI = 23–42) and showed a peculiar tendency to undergo further breakage–fusion cycles as cancers evolved.

For example, we describe *JUN* amplification that originated as a simple reciprocal translocation t(1:10) that underwent further breaking and rejoining (Figure 4B, patient P1) as the primary tumor evolved (P1). Other cases of progressively increased complexity of focal CNAs were noted in *USP14* (P1) and *FGFR1* (P2), which are both involved in cisplatin resistance [15]; *HRAS* oncogene (P2); *SOX2* transcription factor (P5); and *THOC1* (P1), encoding a pro‐apoptotic nuclear matrix protein that induces cell cycle G2 arrest after interacting with Rb [16].

### Osteosarcomas acquire cell cycle G1 mutations no longer than a year before the primary tumor is biopsied

Pairwise comparison of Jaccard similarities derived from CNA data yielded a median value of 0.74 (95% CI = 0.68–0.77; supplementary material, Table S5), suggesting that recurring osteosarcomas developed relatively stable genomes and that most high‐level rearrangements must have existed before the primary tumors were clinically detected. To estimate when these mutations occurred, we used an approximation timing method that was recently applied to similar data from Ewing's sarcomas [17, 18]. In each patient, we first recovered molecular clock signature AC1 (Figure 3) from the primary tumor data; confirmed the steadiness of its increase across sequential tumor samples; and then moved backwards to estimate the time when the tumor emerged. This turned out to be no later than a year prior to the initial biopsy (range 6.9–9.6 months), whereas the divergence of the metastatic cancer cells occurred within 5 months (range 0.6–5.8 months) from the estimated time of origin.

Taking advantage of precomputed 96‐channel SNV mutation signatures, we then searched for ‘genomic scars’ to identify additional biological processes that could be used therapeutically. We specifically recovered aging (AC1), microsatellite‐instability (AC6), and UV exposure signature (AC7), as well as the homologous recombination repair deficiency (HRRD) signature (AC3). The AC3 signature was specifically of interest to our group because it was detected only in four tumors (*n* = 4/12, 33%; P1, P2, and P10), which was fewer than what would be expected from our previous data [19]. To make the present results comparable, we extended our analysis by calculating the level of loss of heterozygosity (LOH‐HDR) [7], telomeric allelic imbalances (TAIs) [9], and large‐scale transitions (LSTs) [8]. Having done so, a higher prevalence of positive patients was achieved, although it varied greatly. For example, testing for LSTs identified 83% (*n* = 10/12) of patients as HRRD‐positive, whereas testing for LOH‐HDRs identified only 42% of cases (*n* = 5/12) (supplementary material, Table S6). However, when all test scores were combined (https://www.accessdata.fda.gov/cdrh_docs/pdf19/P190014B.pdf), only three patients with *BRCA1/BRCA2* mutations remained in the list, plus there was one additional case related to a pathogenic mutation in *PALB2*. To illustrate this case, we outline the clinical course of an 8‐year‐old patient in whom genetically simple primary osteosarcoma recurred as highly rearranged HRRD‐positive metastases after acquiring *PALB2* deletions (supplementary material, Figure S3).

## Discussion

We report that the earliest and thus clinically most promising mutations in osteosarcoma affect the cell cycle G1 transition, which is guarded by cyclins D3, E1, and cyclin‐dependent kinases 2, 4, and 6. Cell cycle G1 alterations originate no more than a year before the primary tumor is clinically detected and occur in a clonally dominant fashion in more than 90% and 50% of patients of the discovery and validation cohorts, respectively.

Our findings suggest that CDK inhibitors might be suitable as an add‐on therapy in osteosarcoma whilst the established treatment protocols remain the backbone therapy for most patients. However, CDK inhibitors are not yet approved for the treatment of bone and soft tissue sarcomas, although the first clinical trial is underway in chordoma (ClinicalTrials.gov Identifier: NCT03110744), another highly malignant tumor of bone. Outside this study, a dramatic response to palbociclib has been reported in a 62‐year‐old patient with advanced refractory CDKN2A‐deficient chordoma who had been surgically treated for seven recurring tumors over the course of 20 years (manuscript under review, personal communication 23 March 2021 from Dr Mrinal Gounder, Department of Medicine, Memorial Sloan Kettering Cancer Center and Weill Cornell Medical College, New York, USA). Since chordomas are known to acquire nearly identical mutation landscapes to osteosarcomas [20], it is plausible that a similar treatment effect of CDK inhibition could be achieved in patients with osteosarcoma.

CDK genes are well expressed in human osteosarcoma tissues and their overexpression correlates with metastatic spread and poor prognosis. In a recent study, Sayles *et al* used palbociclib treatment on patient‐derived osteosarcoma xenografts with *CDK4* amplifications to show that CDK4/6 inhibition interferes with cell cycle progression, induces cell senescence with apoptosis, and decreases the pRb levels [21]. The interaction between CDK4 and Rb is critical for CDK4 inhibitors to work since tumors deficient in Rb are generally insensitive to CDK4 inhibition. Luckily, *RB1* and *CDK4* mutations seem to be mutually exclusive in osteosarcomas and thus CDK4/6 inhibition may be applied without fear of reverting mutations, although they could occur as a response to multimodal chemotherapy. Given that chemotherapy is the current standard line of treatment in osteosarcoma, it is important to add that CDK4 inhibition acts antagonistically to it and therefore strategies positioning CDK4 inhibitors between chemotherapy cycles are being developed to prevent survival of residual tumor cells that escape current chemotherapy regimens.

In addition to *CCND3* amplifications, amplifications of *CCNE1* are common in osteosarcoma as well and the effect of CDK2 inhibition on tumor growth has been demonstrated by dinaciclib (SCH 727965) using patient‐derived xenografts [21]. However, these experiments showed that the current line of CDK2 inhibitors is not entirely specific to CDK2 and therefore more selective inhibitors need to be developed. Targeting CDK2 should therefore be considered with caution but has promise of becoming a therapeutic avenue that might offset tumor resistance to CDK4 inhibition, which *CCNE1* amplifications normally confer to [22].

Cell cycle G1 mutations are typical early mutations but other cancer drivers can occur at any evolutionary time. An interesting finding that falls into this category is mutations that disrupt the ability of a cell to repair DNA damage through homologous recombination (HRR), and we specifically focus on *BRCA2* and *PALB2* mutations because of their association with PARP inhibition sensitivity (PARPi). We note, however, that without *in vitro* experiments the HRR deficiency (HRRD) trait is notoriously difficult to determine and therefore *in silico* tests should be interpreted with caution. For example, our group was the first to describe HRRD in more than 80% of 121 osteosarcomas and our studies are broadly consistent in that HRRD exists in tumors that harbor chromosomal 13 deletions, either as bi‐chromosomal deletions that affect *RB1* and *BRCA2* genes equally or as single‐copy whole‐arm deletions of chromosome 13 that are complemented by focal deletions in *BRCA2*. However, our present and past studies do not yield a similar prevalence of HRRD, due to the use of a more stringent methodology which has been increasingly updated over the last 5 years.

Taken together, our study represents one of the first longitudinal portraits of the osteosarcoma evolution and therefore only a few other studies exist with which to compare our results. In a study analogous to ours, Wang *et al* [23] performed multi‐region sequencing of primary and recurring osteosarcomas of ten patients and used phylogenetic analysis to determine intra‐ and inter‐tumoral heterogeneity. These data confirm that as time progresses, cancer cells acquire more and more mutations with higher antigen load and improved immunogenicity. In both studies, primary tumors were mainly driven by *TP53* mutations, whereas additional drivers such as *BRCA2* mutations occurred mostly in metastatic cells. Metastasis‐specific mutations in osteosarcoma are also described in a study by Suehara *et al* [24], with which our findings are mostly consistent. Both studies describe cancer drivers at similar frequencies and similar tumor manifestations (for example, *BRCA2* mutations in metastases), but Suehara *et al* suggest that co‐amplifications of the regions *PDGFRA–KIT* and *VEGFA–CCND3* are mutually exclusive, which our data contradict.

**Figure 6:**
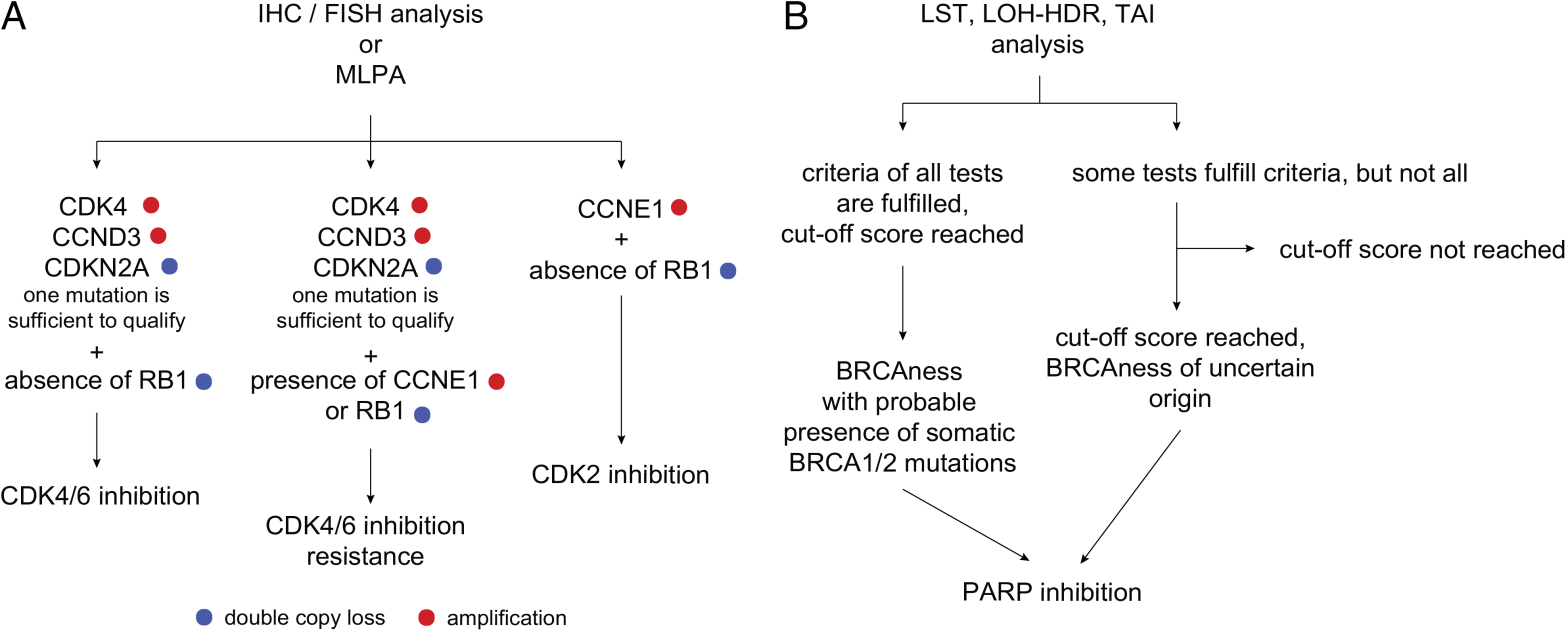
Decision tree for application of CDK and PARP inhibitors. (A) The mutation status of CDK2/4/6, cyclin E1/D3, *RB1*, and *CDKN2A* is determined from sequencing data or by probe‐based assessment techniques (arrays, multiplex ligation‐dependent probe amplification, fluorescence *in situ* hybridization analysis). The tumor's response to CDK4/6 inhibition then depends on fulfilling two conditions: (1) *CCND3* and *CDK4/6* amplifications are present and (2) there are no mutations in *RB1* and *CCNE1*. This is because *CCNE1* amplifications confer CDK4 inhibition resistance by having Rb phosphorylated via the CDK2 pathway and the loss of Rb disrupts CDK4 signal transmission. In the same vein, the success of CDK2 inhibition relies on having the status of cyclin E1 as amplified but Rb status as wild type. (B) The analysis of HRRD follows the Myriad Genetics guidelines: a summary score is calculated from *BRCA1/BRCA2* mutation status and three independent test scores – LST, LOH‐HDR, and TAI. Tumors with cut‐off scores higher than 42 are deemed positive and should respond to PARP inhibition.

Circling back to CDK inhibition, we would like to make one final note. Cell cycle G1 mutations are one of the few targets that are both pervasive and targetable, and activating mutations can be detected in diagnostic biopsies with techniques such as fluorescence *in situ* hybridization (amplifications of *CDK4*, *CCND3*, *CCNE1*), immunohistochemistry (p16 loss), next‐generation sequencing, and multiplex‐ligation probe‐dependent amplification. Assuming that tumor biopsies of the primary osteosarcomas are available, we propose a simple decision tree (Figure 6) to outline the diagnostic algorithm. If primary tumors are not available, the analysis of recurring tumor manifestations should offer an equivalent level of information for clinical staff to decide on an appropriate course of action, as well as providing knowledge about alternative targets such as HRRD mutations.

## Author contributions statement

MK and DB conceived and designed the study. MK, BA, SR, MoK, WC, MB and WH acquired, analyzed or interpreted data. MK, DB and BA drafted the manuscript. MK, DB, BA, MN and WH revised the manuscript for important intellectual content. MN, AK, SB and OW provided administrative, technical or material support. MK and DB supervised the study.

## Supporting information

**Figure S1.***TP53* translocations identified in patients with osteosarcoma
**Figure S2.** Fluorescence *in situ* hybridization analysis shows more than 20 *CDK4* hybridization signals per cell (shown in green), which is well in line with the gene amplification identified from sequencing data of respective patients
**Figure S3.** Phylogenetic and molecular‐genetic analysis of patient P6

**Table S1.** Patients and samples
**Table S2.** Sequencing data summary
**Table S3.** Phylogenetic tree statistics
**Table S4.** Non‐synonymous to synonymous mutation ratios
**Table S5.** Jaccard distances
**Table S6.** Assessments of homologous recombination repair deficiency (HRRD)
