# Supplementary material for: The early evolutionary landscape of osteosarcoma provides clues for targeted treatment strategies
Source: J Pathol. 2021 May 25;254(5):556–66. doi: 10.1002/path.5699 (PMC8361660; doi:10.1002/path.5699)
Supplement: Supplementary file 1 — Figure S1.TP53 translocations identified in patients with osteosarcoma Figure S2. Fluorescence in situ hybridization analysis shows more than 20 CDK4 hybridization signals per cell (shown in green), which is well in line with the gene amplification identified from sequencing data of respective patients Figure S3. Phylogenetic and molecular‐genetic analysis of patient P6 [file PATH-254-556-s002.docx]

**The early evolutionary landscape of osteosarcoma provides clues for targeted treatment strategies**

M Kovac, B Ameline, S Ribi, *et al. J Pathol* DOI: 10.1002/path.5699

**Supplementary Figures S1–S3**


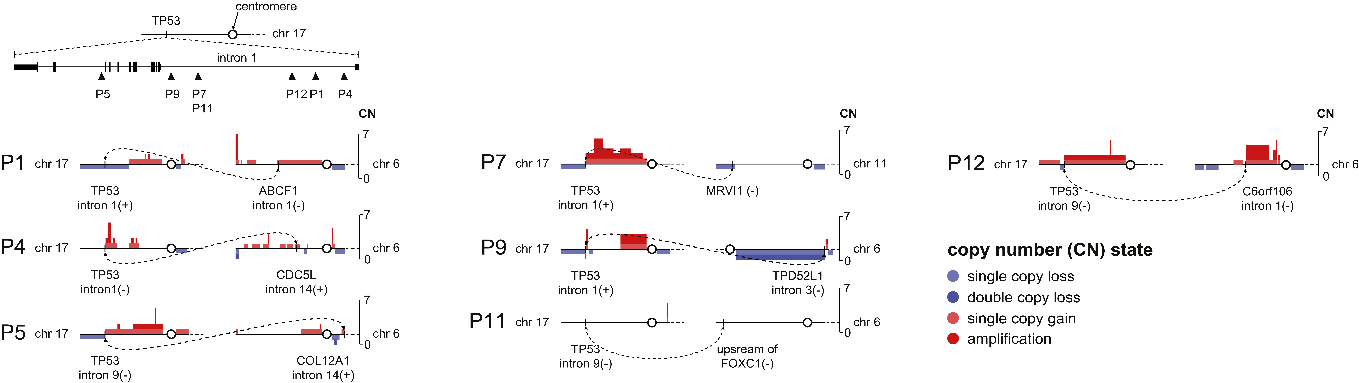


**Figure S1.** *TP53* translocations identified in patients with osteosarcoma. Chromosomal schemas are depicted below copy-number information. Gene names and strand orientation information are depicted below the schema. Note that in *TP53* translocation, partners were from chromosome 6 in 6/7 cases.


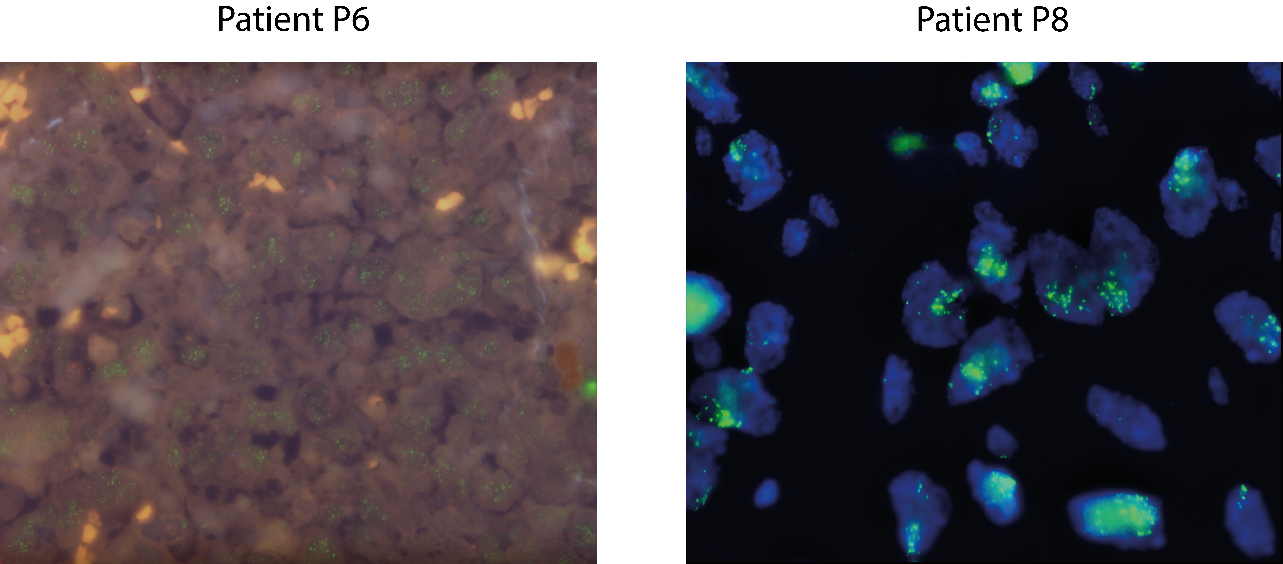


**Figure S2.** Fluorescence *in situ* hybridization analysis shows more than 20 *CDK4* hybridization signals per cell (shown in green), which is well in line with the gene amplification identified from sequencing data of respective patients.


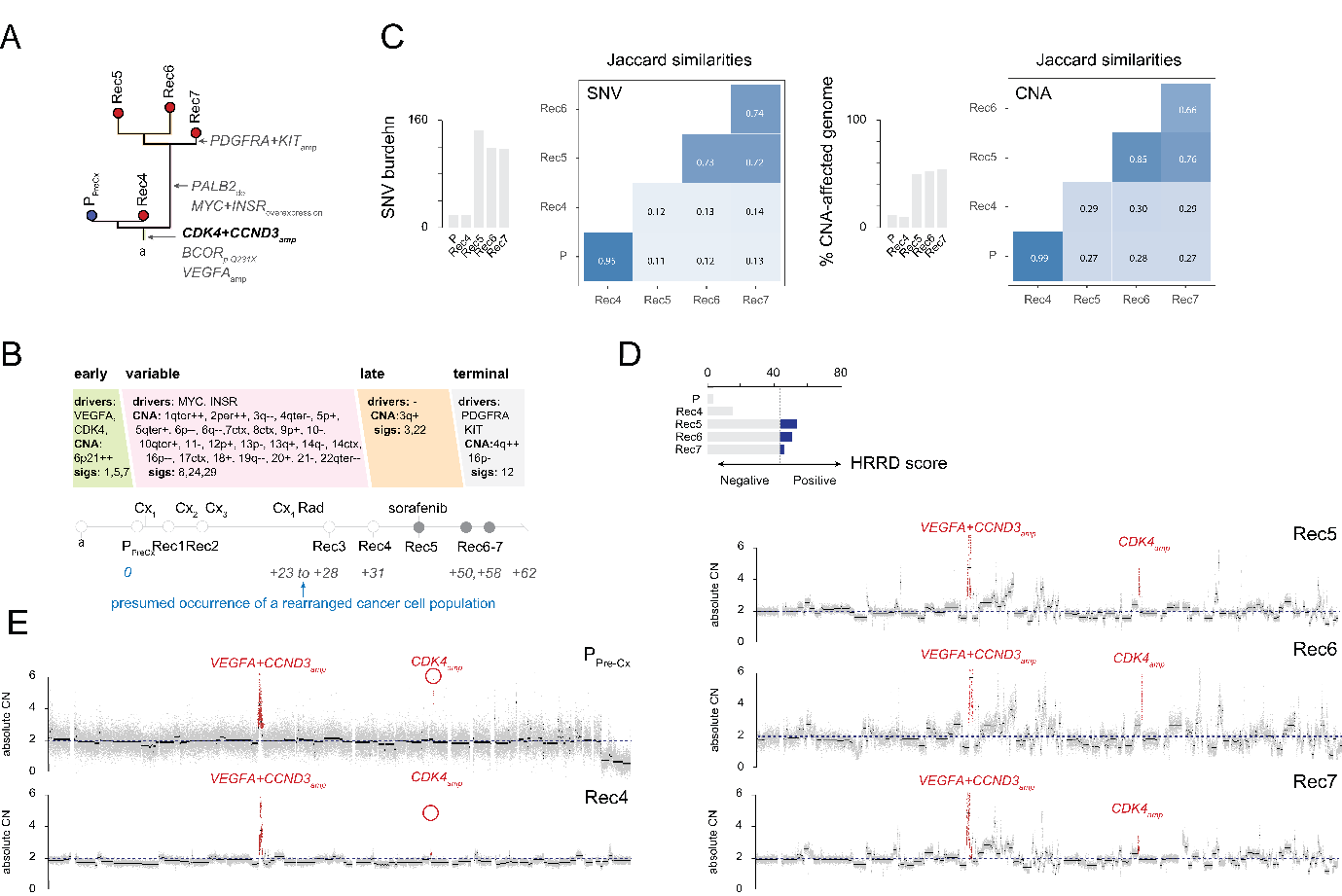


**Figure S3.** Phylogenetic and molecular-genetic analysis of patient P6. (A) Phylogenetic tree. The topology is reminiscent of an unbalanced tree that occurs when some ancestral cancer clone produces more surviving lineage than another, which is a potent indicator of potent sub-clonal selection. (B) Clinical course of the disease that first manifested at the age of 8. The patient underwent a diagnostic biopsy followed by neoadjuvant chemotherapy (EURAMOS1 protocol) before the primary tumor was resected. A first lung metastasis occurred 3 months after surgery. Additional pulmonary recurrences were removed six more times. (C) Jaccard similarity index calculated from SNV and CNA data shows the genetic proximity of two initial tumors (P and Rec4) and three genetically unstable recurrences that occurred after chromoanagenesis (Rec5–7). (D) HRRD assessment of selected tumors. A default threshold of 42 was used as a cut-off value to identify positive metastases. (E) Copy-number profiling demonstrates the punctual evolution of highly rearranged clones that gave rise to the distal recurrences 5–7. Cycle G1 mutations, which are already present in the primary tumor, are highlighted.
